# Appropriate Surrogate Endpoint in Drug-Coated Balloon Trials for Coronary Artery Diseases

**DOI:** 10.3389/fcvm.2022.897365

**Published:** 2022-06-22

**Authors:** Xinyue Lang, Yang Wang, Wei Li, Xiaoyun Liu, Yanyan Zhao, Chuangshi Wang, Xiaocong Li, Yingxuan Zhu, Mengya Li, Lei Song, Bo Xu

**Affiliations:** ^1^Medical Research and Biometrics Center, National Center for Cardiovascular Diseases, Fu Wai Hospital, Chinese Academy of Medical Sciences and Peking Union Medical College, Beijing, China; ^2^Department of Phase I Clinical Trail Center, Beijing Shijitan Hospital, Capital Medical University, Beijing, China; ^3^Department of Cardiology, National Center for Cardiovascular Diseases, Chinese Academy of Medical Sciences, Fu Wai Hospital, Beijing, China

**Keywords:** late lumen loss (LLL), minimal lumen diameter (MLD), percentage diameter stenosis (%DS), drug-coated balloons (DCBs), coronary artery diseases

## Abstract

**Background:**

The appropriateness of using late lumen loss (LLL) as a surrogate endpoint was established in drug-eluting stent (DES) studies, but it was less supportive for drug-coated balloon (DCB) trials.

**Methods:**

Studies published until 23 June 2021 were searched from PubMed, EMBASE, Cochrane Library, and ClinicalTrials.gov. The correlation between LLL, MLD (minimal lumen diameter), and %DS (percentage diameter stenosis) and clinical endpoints was evaluated by linear regression. Standardized effect size and its 95% CIs were used to illustrate the difference among LLL, MLD, and %DS.

**Results:**

A total of 24 clinical trials were eligible [16 DCB vs. DES, 7 DCB vs. plain old balloon angioplasty (POBA), and 1 DCB vs. DES vs. POBA]. Thirteen (54.2%) trials used LLL as the primary endpoint. LLL, MLD, and %DS all had significant associations with clinical endpoints. For DCB vs. DES trials, the number of studies that reported inconsistent results between LLL and MLD was 12/16 (75.0%) and between LLL and %DS was 10/15 (66.7%), while in MLD and %DS, it was 1/16 (6.3%). The difference of standardized effect size between LLL and MLD was −0.47 (95% CI, −0.69 to −0.25, *p* < 0.001) and LLL and %DS was−0.31 (95%CI,−0.43 to−0.20, *p* < 0.001) while in MLD and %DS, there was no difference, 0.1 (95%CI,−0.02 to 0.22, *p* = 0.084).

**Conclusions:**

For DCB trials, an appropriate surrogate is associated with the control device. The traditional LLL could be used in the DCB vs. POBA trials. However, MLD/%DS should be considered a more suitable surrogate endpoint when comparing DCB with DES.

## Introduction

Drug-coated balloons (DCBs) have been extensively used for the treatment of in-stent restenosis (ISR) and *de novo* coronary lesions in some specific settings including small vessel disease (SVD), owing to the antiproliferative drug-eluting capability without the chronic limitations of permanent metallic implants (1). Surrogate endpoints have been widely used to demonstrate the efficacy of DCBs because of feasibility regarding the sample size. Research had proved the robustness of late lumen loss (LLL) in discriminating drug-eluting stent (DES) both in observational and randomized trials (2–4). However, the applicability of LLL to DCB is doubtful.

Late lumen loss is calculated by post-procedure minimal lumen diameter (MLD) minus follow-up MLD, reflecting the narrowing of the luminal diameter immediately after the intervention to the follow-up period. However, due to the elastic retraction, balloon angioplasty has a smaller acute gain compared with permanent scaffolds (5). A small LLL is somehow parallel with a small acute gain (6). In addition, late enlargement and vessel remodeling are achievable in a non-caged vessel in the treatment of DCB (7). Therefore, the surrogate endpoints reflecting the true status of the vessel (e.g. MLD and percentage diameter stenosis (%DS) at follow-up) might be a more ideal choice for DCB trials.

In the current study, we hypothesize that the results between LLL and MLD/%DS may not be consistent. We systematically sorted out the randomized controlled trials (RCT) focusing on DCB, assessed the relationship of these surrogate endpoints with clinical endpoints, and proved the inconsistency of LLL with MLD and %DS.

## Methods

### Study Subjects and Inclusion/Exclusion Criteria

A comprehensive search of studies published before 23 June 2021 was conducted on PubMed, EMBASE, Cochrane Library, and ClinicalTrials.gov, with the search terms (“drug-coated balloon” or “drug-eluting balloon”) and (“late lumen loss” or “late loss” or “late luminal loss” or “minimal lumen diameter” or “minimal luminal diameter” or “diameter stenosis”). All candidate studies were imported into Endnote (Endnote X9.3.1; Thomson Reuters, San Francisco, CA) for screening. The titles and abstracts were checked for possible relevance and duplicated studies were deleted. Further, full articles were reviewed for potential eligibility. Cross-referencing was also used to search for possibly missed articles.

The following inclusion criteria were used to assess the eligibility for each study: (1) in-stent restenosis or de novo coronary lesions; (2) RCT with DCB arm compared with DES or plain old balloon angioplasty (POBA); and (3) report at least two of LLL, MLD, or %DS. The exclusion criteria were as follows: (1) conference abstract, protocol, or review, and (2) the same clinical trial reporting long-term follow-up results.

### Data Extraction

The following study level information was extracted from the recruited trials, including authors, trial name, study design, indication (ISR, *de novo* lesions) (8) interventions, number of patients at baseline, number of patients at follow-up, the definition of the primary endpoint. Further, the key results from each study were collected, including in-segment LLL, in-segement MLD, in-segement %DS, target lesion revascularization (TLR), target lesion failure (TLF), and major adverse cardiovascular events (MACE). For TLR, ischemia-driven TLR (ID-TLR) was preferentially selected. TLF was considered a composite of cardiac death, target vessel myocardial infarction (MI), and TLR. MACE was considered as a composite of any death, MI, and revascularization. When the study reported clinical results for more than one time-point, we chose the timepoint that was closer to the quantitative coronary angiography (QCA) results' timepoint.

### Statistical Analysis

Binary variables were expressed as frequencies with percentages, and continuous variables are expressed as mean ± SD. A 2-sided *p* < 0.05 was considered statistically significant. All analyses were performed using R 4.0.3.

#### Association Between Surrogate and Clinical Endpoints

This part of the analysis was conducted at the interventional group level (the experiment and control arms from one study were used separately). Due to the systematic difference between POBA and the other two devices (DES or DCB), POBA data were excluded from the primary analysis for correlation and regression. Complete analysis (including POBA data) was conducted as sensitivity analysis. Surrogate endpoints included LLL, MLD, and %DS. Clinical endpoints focused on TLR, TLF, and MACE. The correlation coefficients were calculated for each pair of the surrogate and clinical endpoints. Linear regression analyses were performed and standardized regression coefficients (per SD increase) were used to evaluate the reflection of surrogates with the clinical endpoints. The analyses using linear regression models adjusted for the indication (ISR lesions or *de novo* lesions) were also performed. The adjusted β of the surrogate endpoints with clinical endpoints was considered the main indicator of the association. The higher the MLD reflected the better the vessel condition, so the opposite number of MLD was taken to maintain the same direction of benefit with LLL and %DS.

#### Inconsistency in LLL, MLD, and %DS

This part of the analysis was conducted at the trial level. Between-group differences were calculated for LLL, MLD, and %DS within each study. The standardized effect size (*SES*) was created for the comparability of effect size among the above surrogate endpoints. The *SES* was calculated using formula (1) (9, 10), where ${\bar{x}}_{1}$ and ${\bar{x}}_{2}\;$ were the mean of surrogate endpoints for treatment and control, respectively. *n*_1_ and *n*_2_ were the sample size, and *s*_1_ and *s*_2_ represented the standard deviation of each group.

The 95% confidence interval (CI) was calculated by *SES* ± 1.96 *se* where the *se* was calculated using formula (2) (11).

The *SESs* of three surrogate endpoints in each included trial were shown by forest plots grouped by devices: DCB vs. DES or DCB vs. POBA. Because a higher MLD indicates a better status, we took the opposite number of *SES* on MLD to maintain the same direction of benefit with LLL and %DS.


(1) $$\begin{array}{r} SES=\frac{{\bar{x}}_{1}-{\bar{x}}_{2}}{\;}\sqrt{{\frac{\left(n_{1}-1\right){S_{1}}^{2}+\left(n_{2}-1\right)S_{2}}{n_{1}+\;n_{2}-2}}^{2}} \end{array}$$

(2) $$\begin{array}{r} se=\sqrt{\frac{\left(n_{1}+n_{2}-1\right)}{\left(n_{1}+n_{2}-3\right)}\left[\left(\frac{4}{n_{1}+n_{2}}\right)\left(1+\frac{SES^{2}}{8}\right)\right]} \end{array}$$

The inconsistency was considered if two of the surrogate endpoints gave a different result (favored DCB, favored DES, or equal), and the number of the inconsistencies was calculated and presented by *n* (%). Further, the difference in *SES* between every two surrogate endpoints was calculated. The inconsistency was recognized as the opposite direction of the observed effect size (*SES*) among different surrogate endpoints. A paired *t*-test was used to detect the potential discrepancies between LLL, MLD, or %DS. The analysis was grouped by the type of devices (DCB vs. DES/DCB vs. POBA) and compared separately. A subgroup analysis of indication (ISR/*de novo* lesions) was also conducted.

## Results

### Study Selection

The process of the literature search was illustrated in eFigure 1. A total of 1,089 articles were yielded by searching PubMed, EMBASE, Cochrane Library, and ClinicalTrials.gov. A total of 454 articles were excluded owing to duplicates. After reviewing the titles and abstracts, 138 conference abstracts, 5 reviews, 120 protocols, and 286 articles which were irrelevant to our topic were deleted. The remaining 86 studies were further evaluated for eligibility through full texts. Sixty-two articles were further deleted for the following reasons: peripheral artery diseases (*n* = 37), resulting from the same study (*n* = 24), and without a QCA result (*n* = 1). Finally, 24 RCTs were identified, consisting of 16 trials comparing DCB vs. DES (12–27), 7 trials comparing DCB vs. POBA (28–34), and 1 trial comparing DCB vs. DES and POBA (35). The three-arm trial was split into DCB vs. DES and DCB vs. POBA for further analysis.

### Summary of the Included Trials

The 17 recruited trials comparing DCB vs. DES trials involved 3,557 patients, of which 1,822 were allocated to DCB and 1,735 were allocated to DES. The median QCA follow-up rate was 86 and 84% for DCB and DES, respectively. Of the 17 trials, 10 (58.9%) trials were indicated for ISR, and 7 (41.2%) trials were *de novo* lesions. Eight (47.1%) trials used LLL as the primary endpoint, 3 (17.7%) trials used MLD, and 3 (17.7%) trials used %DS (Table 1).

**Table 1:** Baseline information of the included trials.

**References**	**Trial**	**Devices**	**Hypothesis**	**Indication**	**N^**a**^**	**Primary endpoint**
Unverdorben et al. (25)	NA	DCB vs. DES	superiority	ISR	66/65	LLL (6 m)
Byrne et al. (35)	ISAR-DESIRE 3^b^	DCB vs. DES	noninferiority	ISR	137/131	%DS (6–8 m)
Adriaenssens et al. (13)	SEDUCE	DCB vs. DES	not specify	ISR	25/25	uncovered stent struts (9 m)
Alfonso et al. (15)	RIBS V	DCB vs. DES	superiority	ISR	95/94	MLD (6–9 m)
Xu et al. (27)	PEPCAD China ISR	DCB vs. DES	noninferiority	ISR	109/106	LLL (9 m)
Alfonso et al. (14)	RIBS IV	DCB vs. DES	superiority	ISR	154/155	MLD (6–9 m)
Pleva et al. (23)	NA	DCB vs. DES	noninferiority	ISR	68/68	LLL (12 m)
Wong et al. (26)	RESTORE	DCB vs. DES	superiority	ISR	86/86	LLL (9 m)
Baan et al. (16)	DARE	DCB vs. DES	noninferiority	ISR	137/141	MLD (6 m)
Jensen et al. (20)	BIOLUX	DCB vs. DES	noninferiority	ISR	157/72	LLL (6 m)
Cortese et al. (18)	PICCOLETO	DCB vs. DES	noninferiority	*De novo*	29/31	%DS (6 m)
Latib et al. (21)	BELLO	DCB vs. DES	noninferiority	*De novo*	90/92	LLL (6 m)
Tang et al. (24)	RESTORE SVD	DCB vs. DES	noninferiority	*De novo*	116/114	%DS (9 m)
Fahrni et al. (12)	BASKET-SMALL 2	DCB vs. DES	noninferiority	*De novo*	367/371	MACE (12 m)
Cortese et al. (17)	PICCOLETO II	DCB vs. DES	noninferiority	De novo	118/114	LLL (6 m)
Nishiyama et al. (22)	NA	DCB vs. DES	not specify	*De novo*	27/33	8 m
Gobic et al. (19)	NA	DCB vs. DES	not specify	*De novo*	41/37	LLL (6 m)
Habara et al. (29)	NA	DCB vs. POBA	not specify	ISR	25/25	LLL (6 m)
Scheller et al. (32)	PACCOCATH ISR I+II	DCB vs. POBA	superiority	ISR	54/54	LLL (6 m)
Rittger et al. (31)	PEPCAD-DES	DCB vs. POBA	superiority	ISR	72/38	LLL (6 m)
Byrne et al. (35)	ISAR-DESIRE 3	DCB vs. POBA	superiority	ISR	137/134	%DS (6–8 m)
Habara et al. (34)	NA	DCB vs. POBA	superiority	ISR	137/71	TVF (6 m)
Scheller et al. (33)	PATENT-C	DCB vs. POBA	superiority	ISR	33/28	LLL (6 m)
Kleber et al. (30)	NA	DCB vs. POBA	superiority	*De novo*	32/32	LLL (9 m)
Funatsu et al. (28)	NA	DCB vs. POBA	superiority	*De novo*	92/41	TVF (6 m)

^a^*Number of patients*.

^b^*ISAR-DESIRE 3 trial compared DCB with DES and POBA*.

*BMS, bare-mental stents; DCB, drug-coated balloons; DES, drug-eluting stents; %DS, percentage diameter stenosis; HS, healing score; ISR, in-stent restenosis; LLL, late lumen loss; MLD, minimal lumen diameter; MACE, major adverse cardiovascular events; NA, not available; POBA, plain old balloon angioplasty; QCA, quantitative coronary angiography; SV, small vessels; TVF, target vessel failure*.

The 8 recruited studies comparing DCB vs. POBA trials involved 1,005 patients, of which 582 were allocated to DCB, and 423 were allocated to POBA. The median QCA follow-up rate was 88 and 89% among the DCB and POBA groups, respectively. Of the 8 trials, 6 (75.0%) trials were ISR, and 2 (25.0%) trials were *de novo* lesions. Five (62.5%) trials used LLL as the primary endpoint, no trial used MLD as the primary endpoint, and 1 (12.5%) trial used %DS as the primary endpoint (Table 1).

### Association Between LLL, MLD, and %DS vs. Clinical Endpoints

**Figure 1:**
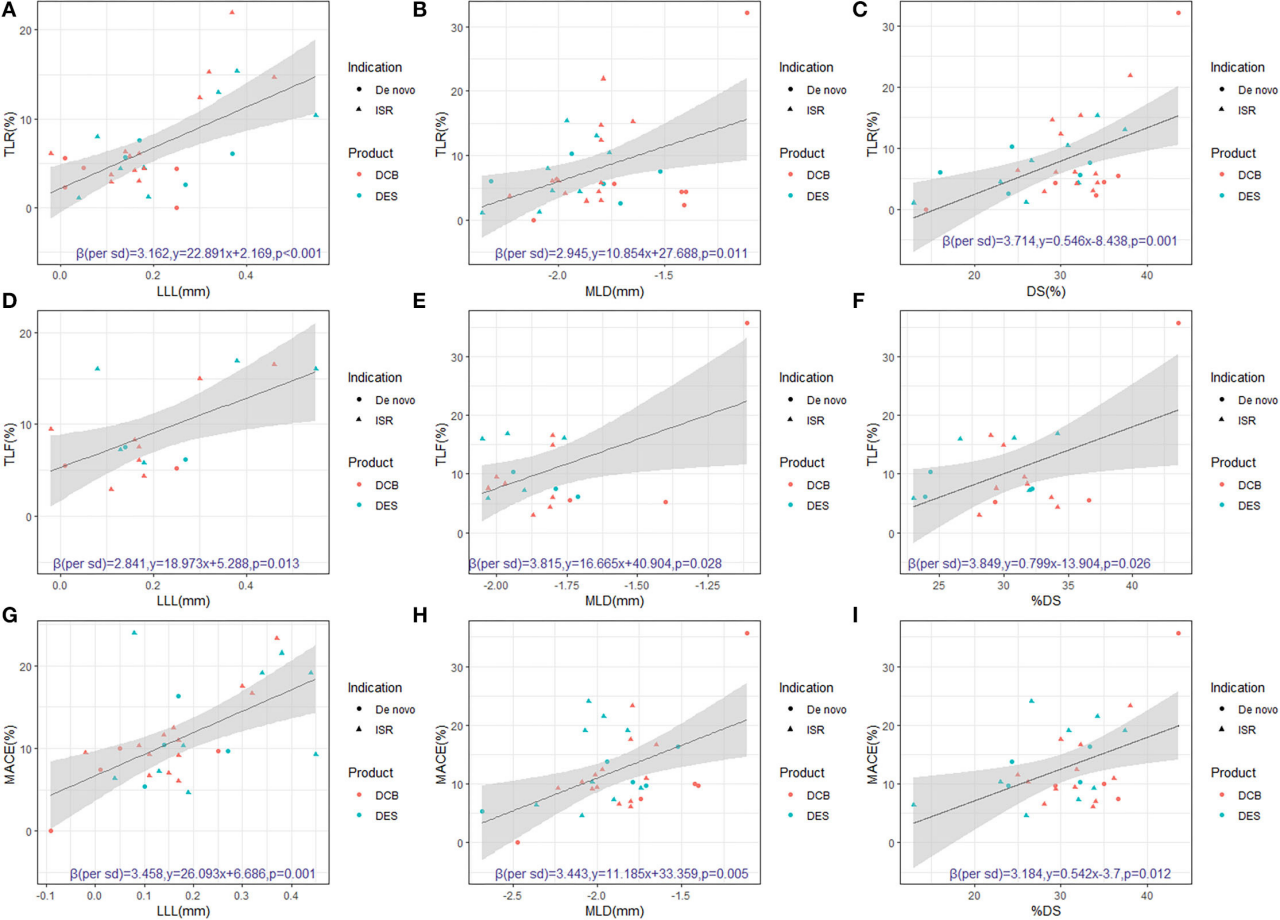
Regression of LLL, MLD, or %DS vs. clinical endpoints (TLR, TLF, and MACE) (Excluding POBA devices). **(A)** For 30 devices reporting the LLL and TLR values, there was a significant relationship between LLL and TLR (R-squared = 0.388 y = 22.891x+2.169, *p* < 0.001). **(B)** For 32 devices reporting the MLD and TLR values, there was a significant relationship between MLD and TLR (R-squared = 0.195, y = 10.854x+27.688, *p* = 0.011). **(C)** For 31 devices reporting the %DS and TLR values, there was a significant relationship between %DS and TLR (R-squared = 0.304, y = 0.546x-8.438, *p* = 0.001). **(D)** For 17 devices reporting the LLL and TLF values, there was a significant relationship between LLL and TLF (R-squared = 0.348, y = 18.973x+5.288, *p* = 0.013). **(E)** For 32 devices reporting the MLD and TLF values, there was a significant relationship between MLD and TLF (R-squared = 0.253, y = 16.665x+40.904, *p* = 0.028). **(F)** For 31 devices reporting the %DS and TLF values, there was a significant relationship between %DS and TLF (R-squared = 0.258, y = 0.799x-13.904, *p* = 0.026). **(G)** For 30 devices reporting the LLL and MACE values, there was a significant relationship between LLL and MACE (R-squared.352, y = 26.093x+6.686, *p* = 0.001). **(H)** For 32 devices reporting the MLD and MACE values, there was a significant relationship between MLD and MACE (R-squared = 0.235, y = 11.185x+33.359, *p* = 0.005). **(I)** For 31 devices reporting the %DS and MACE values, there was a significant relationship between %DS and MACE (R-squared = 0.211, y = 0.542x-3.7, *p* = 0.012). In order to maintain the same direction of benefit, the standardized effect size of MLD here took the opposite number. DCB, drug-coated balloons; DES, drug-eluting stents; %DS, percentage diameter stenosis; ISR, in-stent restenosis; LLL, late lumen loss; MLD, minimal lumen diameter; MACE, major adverse cardiovascular events; POBA, plain old balloon angioplasty; QCA, quantitative coronary angiography; TLF, target lesion failure; TLR, target lesion revascularization.

Among 23 two-arm trials, 20 trials reported three QCA results, 18 trials reported TLR results, 11 trials reported TLF results, and 17 trials reported MACE results and the three-arm trial gave 3 QCA, TLR, and MACE results (eTable 1; eTable 2). All LLL, MLD, and %DS were associated with the incidence of TLR, TLF, and MACE for the included devices and for the subgroup of DCB devices (eTable 3; Figure 1). For DCB devices, after adjusting the indication (ISR or *de novo* lesions), MLD and %DS attributed more clinical endpoints compared with LLL. There were a 6.1% increase in TLR per SD decrease in in-segment MLD but a 4.7% increase in TLR per SD increase in in-segment %DS and a 3.4% increase in TLR per SD increase in in-segment LLL. Similar results were observed on TLF and MACE. For ISR, the LLL, MLD, and %DS showed significant association with TLR and MACE. However, for *de novo* lesions, LLL did not show significant association with TLR, TLF, and MACE, while MLD and %DS showed significant association with TLR and MACE (eFigure 2). Sensitivity analysis (including POBA data) results were shown in the eTable 4, eFigure 3 in (Supplementary Appendix).

### Inconsistency in LLL, MLD, and %DS

**Figure 2:**
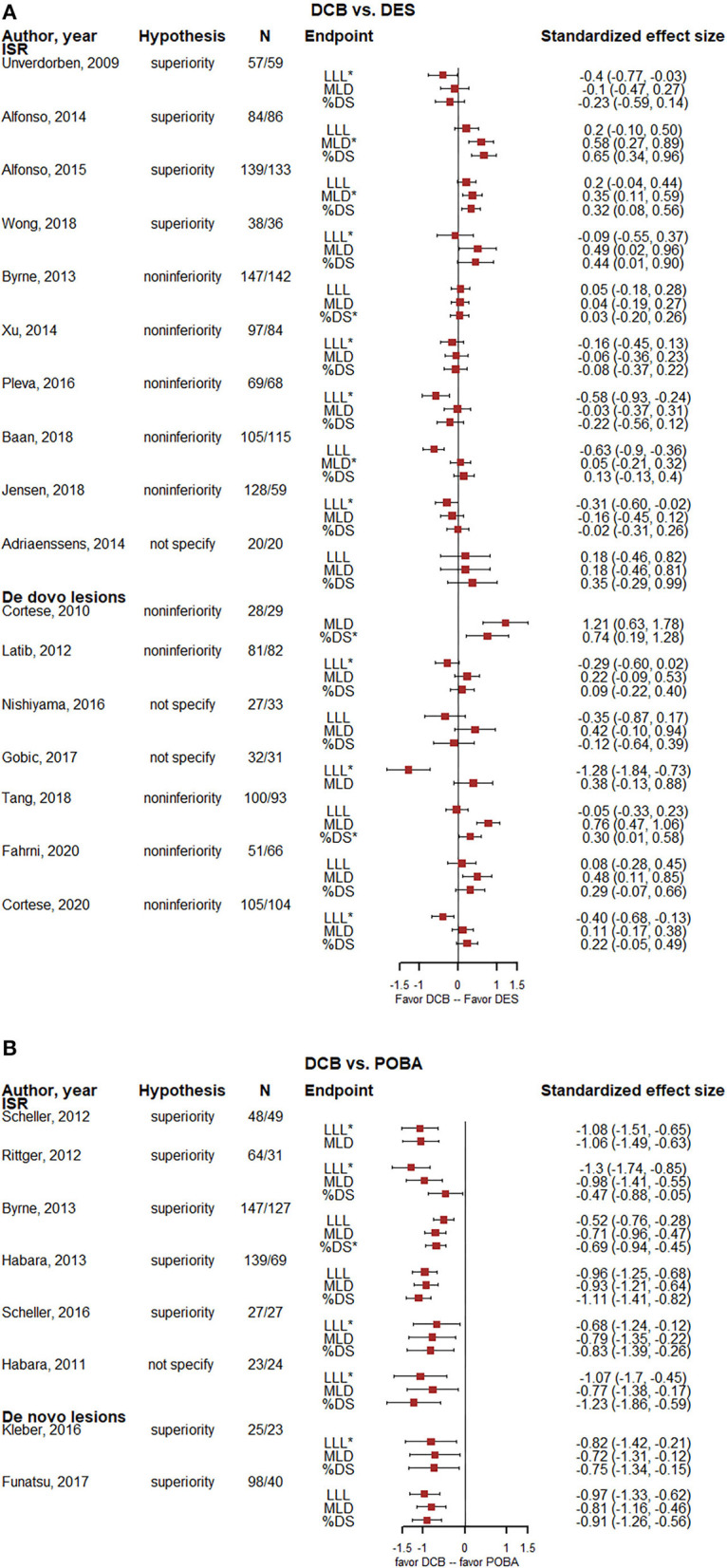
Forest plots of the standardized effect size of LLL, MLD, and %DS. **(A)** Forest plots for 17 DCB vs. DES trials. **(B)** Forest plots for 8 DCB vs. POBA trials. In order to maintain the same direction of benefit, the MLD here took the opposite number. N means the number of lesions. *Means the primary endpoint of the trial. DCB, drug-coated balloons; DES, drug-eluting stents; %DS, percentage diameter stenosis; ISR, in-stent restenosis; LLL, late lumen loss; MLD, minimal lumen diameter; POBA, plain old balloon angioplasty.

The standardized effect size of LLL, MLD, and %DS between the treatment group and control in each recruited study was displayed through forest plots. For trials comparing DCB vs. DES, the number of studies that reported inconsistent results between LLL and MLD was 12/16 (75.0%), between LLL and %DS was 10/15 (66.7%), and between MLD and %DS was 1/16 (6.3%). The subgroup analysis of ISR and *de novo* lesions gave a similar result (Figure 2A; Table 2). The difference of the standardized effect size in LLL and MLD, LLL and %DS were significant, with the means of −0.47 (95%CI, −0.69 to −0.25, *p* < 0.001) and −0.31 (95%CI, −0.43 to −0.20, *p* < 0.001), respectively (Table 2). The subgroup analysis of ISR and *de novo* lesions gave a similar result, except that the standardized effect size of MLD and %DS had a slight difference in *de novo* lesions, 0.28 (95%CI, 0.01 to 0.55, *p* = 0.044).

**Table 2:** Inconsistencies among the surrogate endpoints.

	***N***	**LLL-MLD^**a**^**	***P*-value**	***N***	**LLL-%DS**	***P*-value**	***N***	**MLD-%DS**	***P*-value**
**The number of inconsistencies**, ***n*** **(%)**									
DCB vs. DES									
Total	16	12/16 (75.0)	NA	15	10/15 (66.7)	NA	16	1/16 (6.3)	NA
ISR	10	7/10 (70.0)	NA	10	7/10 (70.0)	NA	10	0/10 (0.0)	NA
*De novo*	6	5/6 (83.3)	NA	5	3/5 (60.0)	NA	6	1/6 (16.7)	NA
DCB vs. POBA									
Total	8	0/8 (0.0)	NA	7	0/7 (0.0)	NA	7	0/7 (0.0)	NA
ISR	6	0/6 (0.0)	NA	5	0/5 (0.0)	NA	5	0/5 (0.0)	NA
*De novo*	2	0/2 (0.0)	NA	2	0/2 (0.0)	NA	2	0/2 (0.0)	NA
**Difference of** ***SES*****, mean (95%CI)**									
DCB vs. DES									
Total	16	−0.47 (−0.69, −0.25)	<0.001	15	−0.31 (−0.43, −0.2)	<0.001	16	0.1 (−0.02, 0.22)	0.084
ISR	10	−0.29 (−0.47, −0.11)	0.006	10	−0.29 (−0.46, −0.12)	0.004	10	0 (−0.08, 0.08)	0.941
*De novo*	6	−0.78 (−1.26, −0.29)	0.009	5	−0.36 (−0.56, −0.15)	0.008	6	0.28 (0.01, 0.55)	0.044
DCB vs. POBA									
Total	8	−0.08 (−0.23, 0.07)	0.259	7	−0.05 (−0.38, 0.29)	0.741	7	0.04 (−0.23, 0.31)	0.726
ISR	6	−0.06 (−0.28, 0.16)	0.504	5	−0.04 (−0.59, 0.51)	0.852	5	0.03 (−0.41, 0.47)	0.854
*De novo*	2	−0.13 (−0.52, 0.26)	0.147	2	−0.07 (−0.12, −0.02)	0.038	2	0.06 (−0.37, 0.5)	0.319

^a^*In order to maintain the same direction of benefit, the standardized effect size of MLD here took the opposite number*.

*CI, confidence interval; DCB, drug-coated balloons; DES, drug-eluting stents; %DS, percentage diameter stenosis; ISR, in-stent restenosis; LLL, late lumen loss; MLD, minimal lumen diameter; NA, not applicable; POBA, plain old balloon angioplasty*.

For trials comparing DCB vs. POBA, no evidence showed discrepancy among the observed effect sizes between LLL, MLD, and %DS (Figure 2B; Table 2). The subgroup analysis of ISR and *de novo* lesions gave a similar result.

## Discussion

Late lumen loss was still the most commonly used surrogate endpoint for DCB in coronary heart disease trials. Our study showed that MLD and %DS, together with LLL, were all correlated with TLR, TLF, and MACE. After adjusting the indication (ISR lesions or *de novo* lesions), MLD and %DS gave more attribution to clinical endpoints compared with LLL. In addition, among studies that compared DCB with POBA, the three QCA surrogate endpoints showed similar observed results. However, for DCB vs. DES trials, the inconsistency of LLL with MLD or %DS was obvious, and the standardized effect sizes in LLL with %DS or MLD were significantly different. However, MLD and %DS gave consistent results and did not have significantly different standardized effect sizes.

The inconsistent observed effect size between LLL and MLD/%DS should be noticed. It indicated that an inappropriate surrogate endpoint may lead to a wrong estimation of the efficacy of DCB. LLL was commonly used as the primary endpoint for DES devices, because the failure of DES was mostly caused by neointimal hyperplasia (36). The validity of LLL had been demonstrated in the era of DES vs. BMS trials (2–4). The acute gain was comparable between experimental and control devices. Therefore, LLL, a direct angiographic measure of neointimal hyperplasia, became an appropriate surrogate endpoint. However, the vessel condition after DCB treatment was different, and the appropriateness of LLL should be re-evaluated accordingly (37–47).

For DCB devices, polymer materials failed to provide effective support equivalent to that of metal stents, which led to acute recoil of blood vessels, and this was the main reason for the restenosis instead of neointimal hyperplasia (7). More importantly, for native vessels, especially in the case of small vessels and distal lesions, the late lumen of some vessels enlarged without reduction (48, 49). The vessels were free from the continuous stimulus caused by the stent, which led to intimal hyperplasia and negative vascular remodeling. Taking the above considerations, the mechanism of changes in vessel diameter was more complex after DCB treatment. The joint effect of acute recoiling, intimal hyperplasia, and vascular remodeling would be attributed to the differences among QCA measurements.

In our study, for DCB vs. DES trials, 12/16 (75%) studies reported inconsistent results between LLL and MLD, and 10/15 (66.7%) studies reported inconsistent results between LLL and %DS, while only one study reported inconsistent results between MLD and %DS. In addition, among the studies which gave an inconsistent QCA result, all LLL indicated better performance of DCB than DES, while MLD and %DS, which directly reflected the state of vessels, did not. Kang et al. (49) conducted a multivariate analysis and demonstrated that post-MLD and %DS were helpful to get optimal results in *de novo* lesions after DCB. It could be partly explained that, after the adjustment of lesion type (ISR or *de novo* lesion), the association between LLL and clinical endpoints were diluted, due to the decision on repeated revascularization which may have systematic difference for patients with or without the stent in their vessel. In general, the relationship with clinical endpoint (TLR, TLF, and MACE) also supported the usage of MLD and %DS as appropriate surrogate endpoints. Thus, we considered that MLD and %DS might be more reliable discriminators of DCB performance.

The strength of our study is that we systematically summarized the relationship between QCA indexes and clinical endpoints in DCB trials and assessed the consistency of LLL, MLD, and %DS using standardized effect sizes.

However, our study has several limitations. First, our study was based on published literature, there were no individual-level patient data. Although we included the RCT trials to partly ensure the quality of recruited studies, besides the LLL, MLD, and %DS, potential other surrogate endpoints such as acute gain were not considered. Second, the period of the eligible studies is > 10 years, and the interventional technology of DES, DCB, and POBA may have undergone major changes. However, according to our inconsistency test of the three QCA indexes, the result did not differ across publication years. Third, the number of eligible studies is not enough for a subgroup analysis (ISR and *de novo* lesions, paclitaxel, and sirolimus-coated balloon). We suggested that the heterogeneity in the mechanism of restenosis after DCB treatment was low. Fourth, the methodology of LLL calculation in DCB trials was inconsistent, which could cause systematic bias. The definition of clinical outcomes was not the same in the original studies. TLR is influenced by both clinical symptoms and QCA results, and using ID-TLR could get a more accurate result (50, 51). Lastly, eligible research lacked intraluminal imaging data, and it is limited to inferring the mechanism of difference between LLL and MLD/%DS based on QCA measurement alone. Therefore, further studies based on patient-level data and with identical definitions of clinical endpoints are still needed to determine the robustness of MLD and %DS for DCB trials.

## Conclusions

For DCB trials, the appropriate surrogate endpoint selection depends on the type of control. The traditional LLL could be used in the DCB vs. POBA trials. However, MLD or %DS should be considered as a more suitable surrogate endpoint instead of LLL when comparing DCB with DES devices.

## Data Availability Statement

The original contributions presented in the study are included in the article/Supplementary Material, further inquiries can be directed to the corresponding authors.

## Author Contributions

XLa analyzed data and wrote the manuscript. YW came up with the conception and design of the paper. CW, XL, YZha, ML, XLi, and YZhu contributed to reviewing, discussing, and amending the manuscript. LS and BX reviewed the manuscript and gave clinical advice. YW and WL did the final review and provided approval of the manuscript. All authors contributed to the article and approved the submitted version.

## Conflict of Interest

The authors declare that the research was conducted in the absence of any commercial or financial relationships that could be construed as a potential conflict of interest.

## Publisher's Note

All claims expressed in this article are solely those of the authors and do not necessarily represent those of their affiliated organizations, or those of the publisher, the editors and the reviewers. Any product that may be evaluated in this article, or claim that may be made by its manufacturer, is not guaranteed or endorsed by the publisher.
