# Supplementary material for: Appropriate Surrogate Endpoint in Drug-Coated Balloon Trials for Coronary Artery Diseases
Source: Front Cardiovasc Med. 2022 Jun 22;9:897365. doi: 10.3389/fcvm.2022.897365 (PMC9256952; doi:10.3389/fcvm.2022.897365)
Supplement: Supplementary file 1 [file Data_Sheet_1.docx]

**Appropriate surrogate endpoint in drug-coated balloons trials for coronary artery diseases**

**(Supplementary Appendix)**

**Supplementary Methods.** Search terms

**eTable 1.** QCA results of the included trials

**eTable 2.** Clinical results of the included trials

**eTable 3.** The correlation between clinical endpoints and QCA endpoints (Excluding POBA devices)

**eTable 4.** The correlation between clinical endpoints and QCA endpoints (Including POBA devices)

**eFigure 1.** Flow Diagram of the Search for Published Studies

**eFigure 2.** Regression of LLL, MLD or %DS versus clinical endpoints (TLR, TLF and MACE) for de novo lesions and ISR (Without POBA devices).

**eFigure 3.** Regression of LLL, MLD or %DS versus clinical results (Including POBA devices).

**Supplementary Methods.** Search terms

**PubMed**

((coated OR eluting) AND balloon)) AND (“late lumen loss” OR “late loss” OR “late luminal loss” OR “minimal lumen diameter” OR “minimal luminal diameter” OR “diameter stenosis”) Filters: **Randomized Controlled Trial**

**Clinical trial.gov**

((coated OR eluting) AND balloon)) AND (“late lumen loss” OR “late loss” OR “late luminal loss” OR “minimal lumen diameter” OR “minimal luminal diameter” OR “diameter stenosis”) | Completed Studies | Studies with Results | Interventional Studies | Adult

**Cochrane library**

((coated OR eluting) AND balloon)) AND (“late lumen loss” OR “late loss” OR “late luminal loss” OR “minimal lumen diameter” OR “minimal luminal diameter” OR “diameter stenosis”)

**Web of science**

#1 TS= ((coated OR eluting) AND balloon)

#2 TS= (“late lumen loss” OR “late loss” OR “late luminal loss” OR “minimal lumen diameter” OR “minimal luminal diameter” OR “diameter stenosis”)

#3 #1 AND #2

**eTable 1.** QCA results of the included trials

| First author, year | Indication | N | LLL (mean ±sd) | | MLD (mean ±sd) | | %DS (mean ±sd) | |
| --- | --- | --- | --- | --- | --- | --- | --- | --- |
|  |  |  | DCB | DES/POBA | DCB | DES/POBA | DCB | DES/POBA |
| DCB vs. DES |  |  |  |  |  |  |  |  |
| Unverdorben, 2009 | ISR | 66/65 | 0.17 ± 0.42 | 0.38 ± 0.61 | 2.03 ± 0.56 | 1.96 ± 0.82 | 29.4 ± 17.5 | 34.2 ± 24.3 |
| Byrne, 2013 | ISR | 137/131 | 0.37 ± 0.59 | 0.34 ± 0.61 | 1.79 ± 0.74 | 1.82 ± 0.74 | 38 ± 21.5 | 37.4 ± 21.8 |
| Adriaenssens, 2014 | ISR | 25/25 | 0.16 ± 0.49 | 0.08 ± 0.4 | 1.97 ± 0.53 | 2.05 ± 0.37 | 31.8 ± 14.9 | 26.6 ± 14.6 |
| Alfonso, 2014 | ISR | 95/94 | 0.14 ± 0.5 | 0.04 ± 0.5 | 2.01 ± 0.6 | 2.36 ± 0.6 | 25 ± 20 | 13 ± 17 |
| Xu, 2014 | ISR | 109/106 | 0.46 ± 0.51 | 0.55 ± 0.61 | 1.8 ± 0.58 | 1.76 ± 0.71 | 29 ± 21.3 | 30.8 ± 25.3 |
| Alfonso, 2015 | ISR | 154/155 | 0.3 ± 0.6 | 0.18 ± 0.6 | 1.8 ± 0.6 | 2.03 ± 0.7 | 30 ± 22 | 23 ± 22 |
| Pleva, 2016 | ISR | 68/68 | 0.09 ± 0.44 | 0.44 ± 0.73 | 2.09 ± 0.57 | 2.07 ± 0.8 | 26.2 ± 18 | 30.9 ± 24.6 |
| Wong, 2018 | ISR | 86/86 | 0.15 ± 0.49 | 0.19 ± 0.41 | 1.8 ± 0.69 | 2.09 ± 0.46 | 34 ± 21 | 26 ± 15 |
| Baan, 2018 | ISR | 137/141 | 0.17 ± 0.41 | 0.45 ± 0.47 | 1.71 ± 0.51 | 1.74 ± 0.61 | 36.1 ± 15.5 | 33.8 ± 18.6 |
| Jensen, 2018 | ISR | 157/72 | -0.02 ± 0.38 | 0.13 ± 0.67 | 2 ± 0.5 | 1.9 ± 0.8 | 31.6 ± 12.8 | 32 ± 23.8 |
| Cortese, 2010 | De novo | 29/31 | NA | NA | 1.11 ± 0.65 | 1.94 ± 0.72 | 43.6 ± 27.4 | 24.3 ± 25.1 |
| Latib, 2012 | De novo | 90/92 | 0.05 ± 0.37 | 0.17 ± 0.45 | 1.42 ± 0.4 | 1.52 ± 0.5 | 35 ± 16 | 33.3 ± 20 |
| Nishiyama, 2016 | De novo | 27/33 | 0.25 ± 0.25 | 0.37 ± 0.4 | 2.12 ± 0.42 | 2.32 ± 0.52 | 14.4 ± 8.3 | 16 ± 15.9 |
| Gobic, 2017 | De novo | 41/37 | -0.09 ± 0.09 | 0.1 ± 0.19 | 2.47 ± 0.5 | 2.68 ± 0.61 | NA | NA |
| Tang, 2018 | De novo | 116/114 | 0.25 ± 0.42 | 0.27 ± 0.36 | 1.4 ± 0.42 | 1.71 ± 0.39 | 29.3 ± 20.2 | 23.9 ± 15.9 |
| Fahrni, 2020 | De novo | 367/371 | 0.1 ± 0.4 | 0.06 ± 0.55 | 1.27 ± 0.4 | 1.49 ± 0.5 | 35.8 ± 20.1 | 29 ± 25.2 |
| Cortese, 2020 | De novo | 118/114 | 0.01 ± 0.25 | 0.14 ± 0.38 | 1.74 ± 0.46 | 1.79 ± 0.48 | 36.6 ± 21 | 32.2 ± 19 |
| DCB vs. POBA |  |  |  |  |  |  |  |  |
| Habara, 2011 | ISR | 25/25 | 0.18 ± 0.45 | 0.72 ± 0.55 | 1.81 ± 0.54 | 1.28 ± 0.8 | 34.2 ± 15.2 | 58 ± 22.7 |
| Scheller, 2012 | ISR | 54/54 | 0.11 ± 0.44 | 0.8 ± 0.79 | 2.23 ± 0.57 | 1.5 ± 0.79 | NA | NA |
| Rittger, 2012 | ISR | 72/38 | 0.32 ± 0.55 | 0.99 ± 0.44 | 1.65 ± 0.66 | 1 ± 0.68 | 32.3 ± 54.7 | 54.7 ± 29.4 |
| Byrne, 2013 | ISR | 137/134 | 0.37 ± 0.59 | 0.7 ± 0.69 | 1.79 ± 0.74 | 1.26 ± 0.75 | 38 ± 21.5 | 54.1 ± 25 |
| Habara, 2013 | ISR | 137/71 | 0.11 ± 0.33 | 0.49 ± 0.5 | 1.87 ± 0.45 | 1.42 ± 0.55 | 28.1 ± 11.1 | 44.1 ± 19.4 |
| Scheller, 2016 | ISR | 33/28 | 0.17 ± 0.4 | 0.48 ± 0.51 | 1.8 ± 0.43 | 1.39 ± 0.6 | 33.7 ± 12.3 | 46.8 ± 18.7 |
| Kleber, 2016 | De novo | 32/32 | 0.08 ± 0.31 | 0.47 ± 0.61 | 1.76 ± 0.36 | 1.37 ± 0.69 | 26.6 ± 12.9 | 42.4 ± 27.5 |
| Funatsu, 2017 | De novo | 92/41 | 0.01 ± 0.31 | 0.32 ± 0.34 | 1.41 ± 0.42 | 1.08 ± 0.37 | 34 ± 14 | 47 ± 15 |

* In order to maintain the same direction of benefit, the standardized difference of MLD here took the opposite number.

Abbreviation: CI, confidence interval; DCB, drug-coated balloons; DES, drug-eluting stents; %DS, percentage diameter stenosis; ISR, in‑stent restenosis; LLL, late lumen loss; MLD, minimal lumen diameter; NA, not available; POBA, plain old balloon angioplasty; QCA, quantitative coronary angiography

**eTable 2.** Clinical results of the included trials

| First author, year | Indication | N | time | TLR n (%) | | | TLF n (%) | | | MACE n (%) | | |
| --- | --- | --- | --- | --- | --- | --- | --- | --- | --- | --- | --- | --- |
|  |  |  |  | DCB | DES/POBA | ID-TLR | DCB | DES/POBA | Definition | DCB | DES/POBA | Definition |
| DCB vs. DES |  |  |  |  |  |  |  |  |  |  |  |  |
| Unverdorben, 2009 | ISR | 66/65 | 12m | 4 (6.1) | 10 (15.4) | No | 5 (7.6) | 11 (16.9) | Cardiac death, MI, stent thrombosis, or TLR | 6 (9.1) | 14 (21.5) | All-cause death, MI, TLR or stent thrombosis |
| Byrne, 2013 | ISR | 137/134 | 12m | 30 (21.9) | 17 (13.0) | No | NA | NA | NA | 32 (23.4) | 25 (19.1) | All-cause death, MI or TLR |
| Adriaenssens, 2014 | ISR | 25/25 | 12m | 1 (4.2) | 2 (8.0) | No | 2 (8.3) | 4 (16.0) | Cardiac death, MI or TLR | 3 (12.5) | 6 (24.0) | All-cause death, MI or TVR |
| Alfonso, 2014 | ISR | 95/94 | 12m | 6 (6.3) | 1 (1.1) | No | NA | NA | NA | 11 (11.6) | 6 (6.4) | Cardiac death, MI or TVR |
| Xu, 2014 | ISR | 109/106 | 12m | 16 (14.7) | 11 (10.4) | Yes | 18 (16.5) | 17 (16.0) | Cardiac death, target vessel MI, or ID-TLR | NA | NA | NA |
| Alfonso, 2015 | ISR | 154/155 | 9m | 19 (12.3) | 7 (4.5) | No | 23 (14.9) | 9 (5.8) | Cardiac death, MI or TLR | 27 (17.5) | 16 (10.3) | All-cause death, MI or TVR |
| Pleva, 2016 | ISR | 68/68 | 12m | NA | NA | NA | NA | NA | NA | 7 (10.3) | 13 (19.1) | Cardiac death, non-fatal acute MI or TVR |
| Wong, 2018 | ISR | 86/86 | 12m | 5 (5.8) | 1 (1.2) | No | NA | NA | NA | 6 (7.0) | 4 (4.7) | All-cause death, MI or TLR |
| Baan, 2018 | ISR | 137/141 | 12m | NA | NA | NA | NA | NA | NA | 15 (10.9) | 13 (9.2) | All-cause death, target vessel MI and TVR. |
| Jensen, 2018 | ISR | 157/72 | 6m | 9 (6.1) | 3 (4.3) | Yes | 14 (9.5) | 5 (7.2) | Cardiac death, target vessel MI, or ID-TLR | 14 (9.5) | 5 (7.2) | Cardiac death, target vessel MI or ID-TLR |
| Cortese, 2010 | De novo | 29/31 | 9m | 9 (32.1) | 3 (10.3) | No | 10 (35.7) | 3 (10.3) | Cardiac death, Q-wave MI or TLR | 10 (35.7) | 4 (13.8) | All-cause death, Q-wave MI, TLR |
| Latib, 2012 | De novo | 90/92 | 6m | 4 (4.4) | 7 (7.6) | No | NA | NA | NA | 9 (10.0) | 15 (16.3) | All-cause death, Q- or non–Q-wave MI, or TVR |
| Nishiyama, 2016 | De novo | 27/33 | 9m | 0 (0.0) | 2 (6.1) | No | NA | NA | NA | NA | NA | NA |
| Gobic, 2017 | De novo | 41/37 | 6m | NA | NA | NA | NA | NA | NA | 0 (0.0) | 2 (5.4) | Cardiac death, reinfarction, TLR and stent thrombosis. |
| Tang, 2018 | De novo | 116/114 | 12m | 5 (4.4) | 3 (2.6) | Yes | 6 (5.3) | 7 (6.1) | Cardiac death, target vessel MI, or ID-TLR | 11 (9.6) | 11 (9.6) | All-cause death, all MI, or any revascularization. |
| Fahrni, 2020 | De novo | 367/371 | NA | NA | NA | NA | NA | NA | NA | NA | NA | NA |
| Cortese, 2020 | De novo | 118/114 | 12m | 6 (5.6) | 6 (5.7) | No | 6 (5.6) | 8 (7.5) | Cardiac death, MI or TLR | 8 (7.4) | 11 (10.4) | All-cause death, MI, vessel thrombosis or TLR |
| DCB vs. POBA |  |  |  |  |  |  |  |  |  |  |  |  |
| Habara, 2011 | ISR | 25/25 | 6m | 1 (4.3) | 10 (41.7) | No | 1 (4.3) | 10 (41.7) | Cardiac death, target vessel MI or TLR | NA | NA | NA |
| Scheller, 2012 | ISR | 54/54 | 12m | 2 (3.7) | 20 (37.0) | No | NA | NA | NA | 5 (9.3) | 24 (44.4) | All-cause death, MI, stroke or TLR |
| Rittger, 2012 | ISR | 72/38 | 6m | 11 (15.3) | 14 (36.8) | No | NA | NA | NA | 12 (16.7) | 19 (50.0) | All-cause death, MI or TLR |
| Byrne, 2013 | ISR | 137/134 | 12m | 30 (21.9) | 56 (41.8) | No | NA | NA | NA | 32 (23.4) | 61 (45.5) | All-cause death, MI or TLR |
| Habara, 2013 | ISR | 137/71 | 6m | 4 (2.9) | 22 (31.0) | No | 4 (2.9) | 22 (31.0) | Cardiac death, MI or TLR | 9 (6.6) | 22 (31.0) | All-cause death, nonfatal repeat acute MI, stent thrombosis, or TVF |
| Scheller, 2016 | ISR | 33/28 | 6m | 1 (3.0) | 9 (32.1) | Yes | 2 (6.1) | 9 (32.1) | Cardiac death, target vessel MI, or ID-TLR | 2 (6.1) | 9 (32.1) | Cardiac death, target vessel MI, or ID-TLR |
| Kleber, 2016 | De novo | 32/32 | NA | NA | NA | NA | NA | NA | NA | NA | NA | NA |
| Funatsu, 2017 | De novo | 92/41 | 12m | 2 (2.3) | 4 (10.3) | No | NA | NA | NA | NA | NA | NA |

* In order to maintain the same direction of benefit, the standardized difference of MLD here took the opposite number.

Abbreviation: DCB, drug-coated balloons; DES, drug-eluting stents; %DS, percentage diameter stenosis; ISR, in‑stent restenosis; ID-TLR, ischemia-driven TLR; LLL, late lumen loss; MLD, minimal lumen diameter; MACE, major adverse cardiovascular events; NA, not available; MI, myocardial infarction; POBA, plain old balloon angioplasty; QCA, quantitative coronary angiography; TLF, target lesion failure; TLR, target lesion revascularization; TVF, target vessel failure; TVR, target vessel revascularization;

**eTable 3.** The correlation between clinical endpoints and QCA endpoints (Excluding POBA devices)

|  |  | LLL | MLD ^a^ | %DS |
| --- | --- | --- | --- | --- |
| TLR |  |  |  |  |
| Total | N | 30 | 32 | 31 |
|  | *r* | 0.469, p = 0.009 | 0.270, p = 0.136 | 0.386, p = 0.032 |
|  | *β* (per sd) | 3.162, p < 0.001 | 2.945, p = 0.011 | 3.714, p = 0.001 |
|  | *Adjusted β* (per sd) | 2.978, p < 0.001 | 3.796, p = 0.003 | 3.700, p = 0.002 |
| DES | N | 12 | 13 | 13 |
|  | *r* | 0.538, p = 0.071 | 0.297, p = 0.325 | 0.626, p = 0.022 |
|  | *β* (per sd) | 2.698, p = 0.039 | 1.395, p = 0.293 | 2.703, p = 0.026 |
|  | *Adjusted β* (per sd) | 2.702, p = 0.045 | 1.666, p = 0.249 | 2.696, p = 0.036 |
| DCB | N | 18 | 19 | 18 |
|  | *r* | 0.420, p = 0.083 | 0.303, p = 0.207 | 0.261, p = 0.295 |
|  | *β* (per sd) | 3.788, p = 0.002 | 3.823, p = 0.036 | 4.628, p = 0.013 |
|  | *Adjusted β* (per sd) | 3.420, p = 0.006 | 6.100, p = 0.007 | 4.658, p = 0.016 |
| TLF |  |  |  |  |
| Total | N | 17 | 19 | 19 |
|  | *r* | 0.355, p = 0.163 | -0.078, p = 0.75 | 0.104, p = 0.673 |
|  | *β* (per sd) | 2.841, p = 0.013 | 3.815, p = 0.028 | 3.849, p = 0.026 |
|  | *Adjusted β* (per sd) | 2.634, p = 0.018 | 5.313, p = 0.016 | 3.815, p = 0.034 |
| DES | N | 7 | 8 | 8 |
|  | *r* | 0.321, p = 0.482 | -0.143, p = 0.736 | 0.643, p = 0.086 |
|  | *β* (per sd) | 2.518, p = 0.270 | -1.115, p = 0.581 | 2.052, p = 0.292 |
|  | *Adjusted β* (per sd) | 2.113, p = 0.347 | 0.086, p = 0.97 | 1.513, p = 0.455 |
| DCB | N | 10 | 11 | 11 |
|  | *r* | 0.292, p = 0.413 | 0.147, p = 0.667 | 0.173, p = 0.612 |
|  | *β* (per sd) | 2.829, p = 0.049 | 6.147, p = 0.028 | 6.061, p = 0.031 |
|  | *Adjusted β* (per sd) | 2.657, p = 0.077 | 10.105, p = 0.028 | 6.331, p = 0.072 |
| MACE |  |  |  |  |
| Total | N | 30 | 32 | 29 |
|  | *r* | 0.474, p = 0.008 | 0.333, p = 0.063 | 0.281, p = 0.14 |
|  | *β* (per sd) | 3.458, p = 0.001 | 3.443, p = 0.005 | 3.184, p = 0.012 |
|  | *Adjusted β* (per sd) | 3.245, p = 0.002 | 3.749, p = 0.003 | 3.149, p = 0.016 |
| DES | N | 13 | 14 | 13 |
|  | *r* | 0.264, p = 0.384 | 0.257, p = 0.375 | 0.434, p = 0.138 |
|  | *β* (per sd) | 2.356, p = 0.23 | 2.01, p = 0.269 | 2.709, p = 0.137 |
|  | *Adjusted β* (per sd) | 2.111, p = 0.316 | 2.199, p = 0.24 | 2.709, p = 0.157 |
| DCB | N | 17 | 18 | 16 |
|  | *r* | 0.565, p = 0.018 | 0.417, p = 0.085 | 0.226, p = 0.399 |
|  | *β* (per sd) | 4.21, p < 0.001 | 4.906, p = 0.005 | 4.566, p = 0.014 |
|  | *Adjusted β* (per sd) | 4.048, p < 0.001 | 5.639, p = 0.004 | 4.737, p = 0.027 |

^a^ In order to maintain the same direction of benefit, the standardized effect size of MLD here took the opposite number.

Adjusted for indication (ISR or de novo lesions).

Abbreviation: DCB, drug-coated balloons; DES, drug-eluting stents; %DS, percentage diameter stenosis; ISR, in‑stent restenosis; LLL, late lumen loss; MLD, minimal lumen diameter; MACE, major adverse cardiovascular events; POBA, plain old balloon angioplasty; QCA, quantitative coronary angiography; TLF, target lesion failure; TLR, target lesion revascularization.

**eTable 4.** The correlation between clinical endpoints and QCA endpoints (Including POBA devices)

|  |  | LLL | MLD * | %DS |
| --- | --- | --- | --- | --- |
| TLR |  |  |  |  |
| Total | N | 37 | 39 | 37 |
|  | *r* | 0.708, p < 0.001 | 0.528, p = 0.001 | 0.608, p < 0.001 |
|  | *β* (per sd) | 10.601, p < 0.001 | 8.064, p < 0.001 | 9.661, p < 0.001 |
|  | *Adjusted β* (per sd) | 10.200, p < 0.001 | 9.224, p < 0.001 | 9.425, p < 0.001 |
| DES | N | 12 | 13 | 13 |
|  | *r* | 0.538, p = 0.071 | 0.297, p = 0.325 | 0.626, p = 0.022 |
|  | *β* (per sd) | 2.698, p = 0.039 | 1.395, p = 0.293 | 2.703, p = 0.026 |
|  | *Adjusted β* (per sd) | 2.702, p = 0.045 | 1.666, p = 0.249 | 2.696, p = 0.036 |
| DCB | N | 18 | 19 | 18 |
|  | *r* | 0.420, p = 0.083 | 0.303, p = 0.207 | 0.261, p = 0.295 |
|  | *β* (per sd) | 3.788, p = 0.002 | 3.823, p = 0.036 | 4.628, p = 0.013 |
|  | *Adjusted β* (per sd) | 3.42, p = 0.006 | 6.100, p = 0.007 | 4.658, p = 0.016 |
| POBA | N | 7 | 7 | 6 |
|  | *r* | 0.607, p = 0.148 | -0.036, p = 0.939 | 0.657, p = 0.156 |
|  | *β* (per sd) | 8.033, p = 0.057 | -3.487, p = 0.482 | 7.574, p = 0.165 |
|  | *Adjusted β* (per sd) | 2.884, p = 0.270 | 1.626, p = 0.505 | 4.462, p = 0.026 |
| TLF |  |  |  |  |
| Total | N | 20 | 22 | 22 |
|  | *r* | 0.592, p = 0.006 | 0.287, p = 0.195 | 0.420, p = 0.052 |
|  | *β* (per sd) | 8.587, p < 0.001 | 8.055, p < 0.001 | 9.207, p < 0.001 |
|  | *Adjusted β* (per sd) | 8.207, p < 0.001 | 9.512, p < 0.001 | 9.182, p < 0.001 |
| DES | N | 7 | 8 | 8 |
|  | *r* | 0.321, p = 0.482 | -0.143, p = 0.736 | 0.643, p = 0.086 |
|  | *β* (per sd) | 2.518, p = 0.270 | -1.115, p = 0.581 | 2.052, p = 0.292 |
|  | *Adjusted β* (per sd) | 2.113, p = 0.347 | 0.086, p = 0.97 | 1.513, p = 0.455 |
| DCB | N | 10 | 11 | 11 |
|  | *r* | 0.292, p = 0.413 | 0.147, p = 0.667 | 0.173, p = 0.612 |
|  | *β* (per sd) | 2.829, p = 0.049 | 6.147, p = 0.028 | 6.061, p = 0.031 |
|  | *Adjusted β* (per sd) | 2.657, p = 0.077 | 10.105, p = 0.028 | 6.331, p = 0.072 |
| POBA | N | 3 | 3 | 3 |
|  | *r* | 0.500, p = 0.667 | 1.000, p < 0.001 | 1.000, p < 0.001 |
|  | *β* (per sd) | 5.807, p = 0.086 | 5.828, p = 0.068 | 5.837, p = 0.054 |
| MACE |  |  |  |  |
| Total | N | 35 | 37 | 33 |
|  | *r* | 0.669, p < 0.001 | 0.551, p < 0.001 | 0.511, p = 0.002 |
|  | *β* (per sd) | 10.746, p < 0.001 | 8.459, p < 0.001 | 9.031, p < 0.001 |
|  | *Adjusted β* (per sd) | 10.653, p < 0.001 | 8.655, p < 0.001 | 8.988, p < 0.001 |
| DES | N | 13 | 14 | 13 |
|  | *r* | 0.264, p = 0.384 | 0.257, p = 0.375 | 0.434, p = 0.138 |
|  | *β* (per sd) | 2.356, p = 0.230 | 2.010, p = 0.269 | 2.709, p = 0.137 |
|  | *Adjusted β* (per sd) | 2.111, p = 0.316 | 2.199, p = 0.240 | 2.709, p = 0.157 |
| DCB | N | 17 | 18 | 16 |
|  | *r* | 0.565, p = 0.018 | 0.417, p = 0.085 | 0.226, p = 0.399 |
|  | *β* (per sd) | 4.210, p < 0.001 | 4.906, p = 0.005 | 4.566, p = 0.014 |
|  | *Adjusted β* (per sd) | 4.048, p < 0.001 | 5.639, p = 0.004 | 4.737, p = 0.027 |
| POBA | N | 5 | 5 | 4 |
|  | *r* | 0.800, p = 0.104 | 0.700, p = 0.188 | 1.000, p < 0.001 |
|  | *β* (per sd) | 8.099, p = 0.014 | 5.321, p = 0.261 | 9.320, p = 0.023 |

* In order to maintain the same direction of benefit, the standardized difference of MLD here took the opposite number.

Adjusted for indication (ISR or de novo lesions).

Abbreviation: DCB, drug-coated balloons; DES, drug-eluting stents; %DS, percentage diameter stenosis; ISR, in‑stent restenosis; LLL, late lumen loss; MLD, minimal lumen diameter; MACE, major adverse cardiovascular events; POBA, plain old balloon angioplasty; QCA, quantitative coronary angiography; TLF, target lesion failure; TLR, target lesion revascularization.

**Web of Science**

**34**

**Clinical trial.gov**

**67**

**PubMed**

**334**

**Cochrane Library**

**654**

**Total: 1089**

**Duplicates: 454**

**Titles/abstracts screened: 635**

**138: Conference abstract**

**5: Review**

**120: Protocol**

**286: Irrelevance to our topic**

**Full-text articles assessed: 86**

**37:** **peripheral artery disease**

**24: result from same study**

**1:** **without QCA result**

**24 original articles included:**

**16:** **DCB vs. DES**

**7: DCB vs. POBA**

**1: DCB vs. DES vs. POBA**

**eFigure 1. Flow Diagram of the Search for Published Studies**

Abbreviation: DCB, drug-coated balloons; DES, drug-eluting stents; POBA, plain old balloon angioplasty; QCA, quantitative coronary angiography.


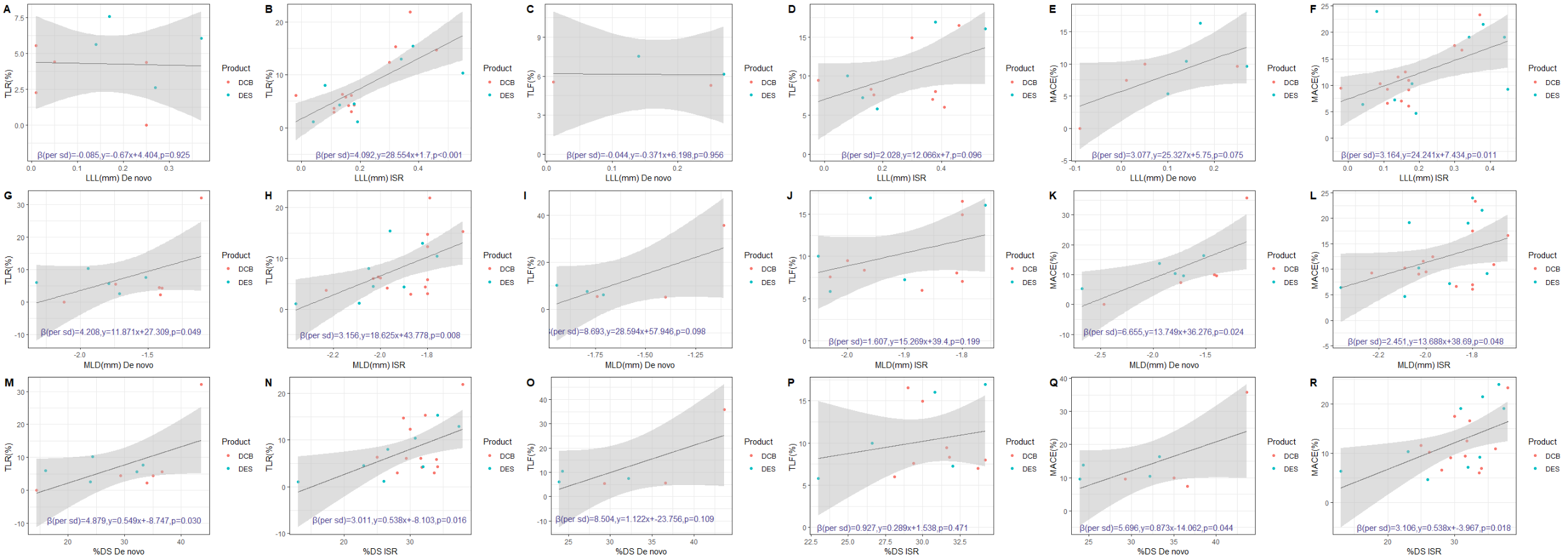
**eFigure 2. Regression of LLL, MLD or %DS versus clinical endpoints (TLR, TLF and MACE) for de novo lesions and ISR (Without POBA devices).**

A. For 9 devices reporting LLL and TLR value for de novo lesions, there was no relationship between LLL and TLR (R-squared=0.001, y=-0.67x+4.404, p=0.925). B. For 21 devices reporting LLL and TLR value for ISR, there was a significant relationship between LLL and TLR (R-squared=0.535, y=28.554x+1.7, p<0.001). C. For 4 devices reporting LLL and TLF value for de novo lesions, there was no relationship between LLL and TLF (R-squared=0.002, y=-0.371x+6.198, p=0.956). D. For 13 devices reporting LLL and TLF value for ISR, there was no significant relationship between LLL and TLF (R-squared=0.232, y=12.066x+7, p=0.096). E. For 8 devices reporting LLL and MACE value for de novo lesions, there was no significant relationship between LLL and MACE (R-squared=0.435, y=25.327x+5.75, p=0.075). F. For 22 devices reporting LLL and MACE value for ISR, there was a significant relationship between LLL and MACE (R-squared=0.28, y=24.241x+7.434, p=0.011). G. For 11 devices reporting MLD and TLR value for de novo lesions, there was significant relationship between MLD and TLR (R-squared=0.237, y=11.871x+27.309, p=0.049). H. For 21 devices reporting MLD and TLR value for ISR, there was a significant relationship between MLD and TLR (R-squared=0.318, y=18.625x+43.778, p=0.008). I. For 6 devices reporting MLD and TLF value for de novo lesions, there was no significant relationship between MLD and TLF (R-squared=0.536, y=28.594x+57.946, p=0.098). J. For 13 devices reporting MLD and TLF value for ISR, there was no relationship between MLD and TLF (R-squared=0.334, y=15.269x+39.4, p=0.199). K. For 10 devices reporting MLD and MACE value for de novo lesions, there was a significant relationship between MLD and MACE (R-squared=0.492, y=13.749x+36.276, p=0.024). L. For 22 devices reporting MLD and MACE value for ISR, there was a significant relationship between MLD and MACE (R-squared=0.168, y=13.688x+38.69, p=0.048). M. For 11 devices reporting %DS and TLR value for de novo lesions, there was a significant relationship between %DS and TLR (R-squared=0.319, y=0.549x+-8.747, p=0.030). N. For 20 devices reporting %DS and TLR value for ISR, there was a significant relationship between %DS and TLR (R-squared=0.283, y=0.538x+-8.103, p=0.016). O. For 6 devices reporting %DS and TLF value for de novo lesions, there was no relationship between %DS and TLF (R-squared=0.513, y=1.122x+-23.756, p=0.109). P. For 13 devices reporting %DS and TLF value for ISR, there was no relationship between %DS and TLF (R-squared=0.048, y=0.289x+1.538, p=0.471). Q. For 8 devices reporting %DS and MACE value for de novo lesions, there was a significant relationship between %DS and MACE (R-squared=0.387, y=0.873x-14.062, p=0.044). R. For 21 devices reporting %DS and MACE value for ISR, there was a significant relationship between %DS and MACE (R-squared=0.261, y=0.538x-3.967, p=0.018).

In order to maintain the same direction of benefit, the standardized difference of MLD here took the opposite number.

Abbreviation: DCB, drug-coated balloons; DES, drug-eluting stents; %DS, percentage diameter stenosis; ISR, in‑stent restenosis; LLL, late lumen loss; MLD, minimal lumen diameter; MACE, major adverse cardiovascular events; POBA, plain old balloon angioplasty; QCA, quantitative coronary angiography; TLF, target lesion failure; TLR, target lesion revascularization.


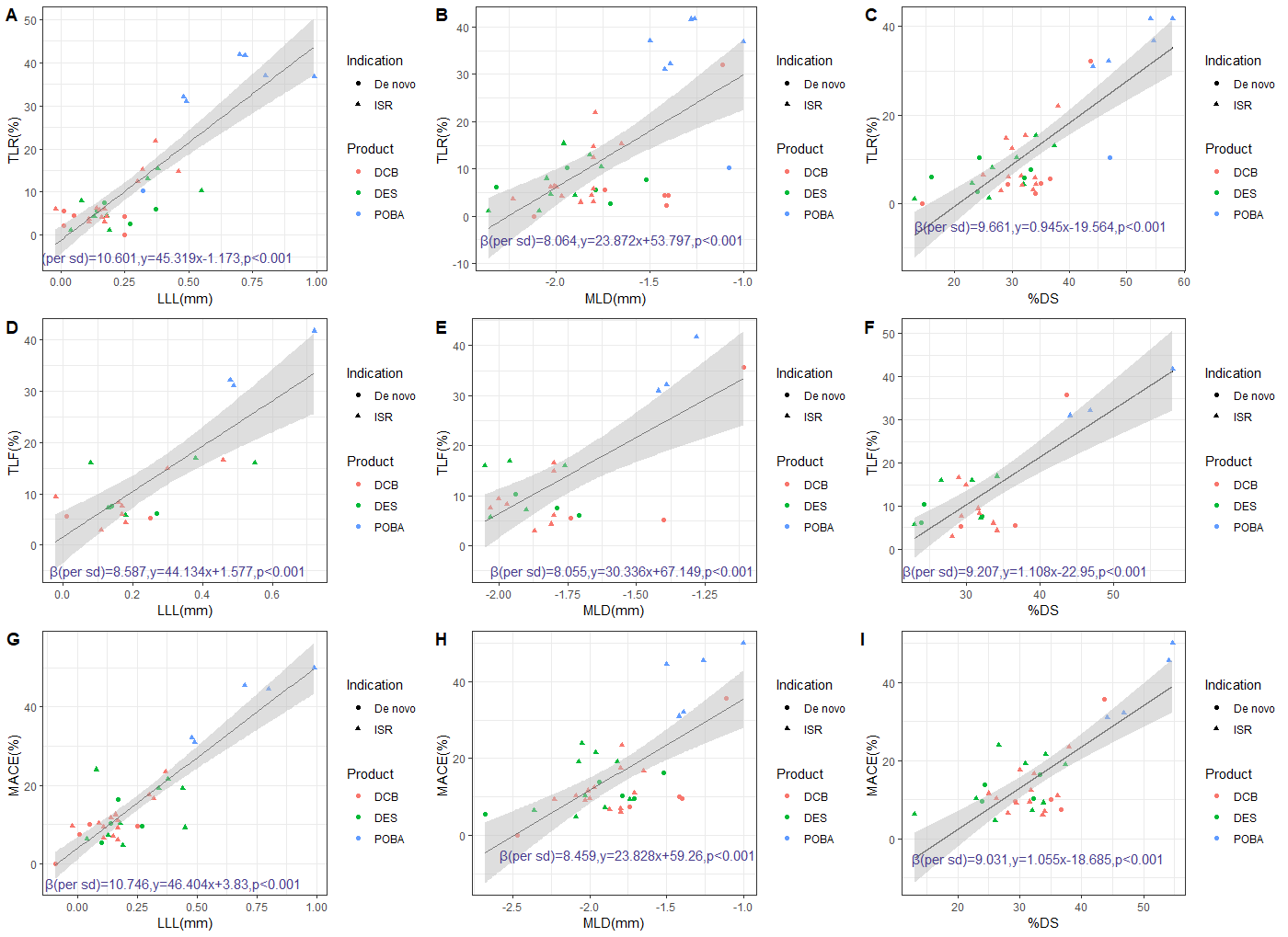


**eFigure 3. Regression of LLL, MLD or %DS versus clinical endpoints (TLR, TLF and MACE) (Including POBA devices).** A. For 37 devices reporting LLL and TLR value, there was a significant relationship between LLL and TLR (R-squared=0.757, y=45.319x+-1.173, p < 0.001). B. For 39 devices reporting MLD and TLR value, there was a significant relationship between MLD and TLR (R-squared=0.429, y=23.872x+53.797, p < 0.001). C. For 37 devices reporting %DS and TLR value, there was a significant relationship between %DS and TLR (R-squared=0.664, y=0.945x-19.564, p < 0.001). D. For 20 devices reporting LLL and TLF value, there was a significant relationship between LLL and TLF (R-squared=0.659, y=44.134x+1.577, p < 0.001). E. For 22 devices reporting MLD and TLF value, there was a significant relationship between MLD and TLF (R-squared=0.518, y=30.336x+67.149, p < 0.001). F. For 22 devices reporting %DS and TLF value, there was a significant relationship between %DS and TLF (R-squared=0.677, y=1.108x-22.95, p < 0.001). G. For 35 devices reporting LLL and MACE value, there was a significant relationship between LLL and MACE (R-squared=0.794, y=46.404x+3.83, p < 0.001). H. For 37 devices reporting MLD and MACE value, there was a significant relationship between MLD and MACE (R-squared=0.482, y=23.828x+59.26, p < 0.001). I. For 33 devices reporting %DS and MACE value, there was a significant relationship between %DS and MACE (R-squared=0.633, y=1.055x-18.685, p < 0.001).

In order to maintain the same direction of benefit, the standardized difference of MLD here took the opposite number.

Abbreviation: DCB, drug-coated balloons; DES, drug-eluting stents; %DS, percentage diameter stenosis; ISR, in‑stent restenosis; LLL, late lumen loss; MLD, minimal lumen diameter; MACE, major adverse cardiovascular events; POBA, plain old balloon angioplasty; QCA, quantitative coronary angiography; TLF, target lesion failure; TLR, target lesion revascularization.
